# Exploring the Relationship Between Diabetes and Breast Cancer in the United Arab Emirates

**DOI:** 10.7759/cureus.54787

**Published:** 2024-02-23

**Authors:** Fatima Alharmoodi, Mouza A Al Ameri, Mohammed Alblooshi, Nandan M Shanbhag, Mariam H Almheiri, Abdulrahman Bin Sumaida

**Affiliations:** 1 General Surgery, Tawam Hospital, Al Ain, ARE; 2 Oncology, Tawam Hospital, Al Ain, ARE; 3 Medicine and Health Sciences, United Arab Emirates University, Al Ain, ARE; 4 Oncology/Radiation Oncology, Tawam Hospital, Al Ain, ARE; 5 Oncology/Palliative Care, Tawam Hospital, Al Ain, ARE

**Keywords:** comorbidities, oncology, united arab emirates, breast cancer, diabetes

## Abstract

Introduction

This study delves into the complex interplay between diabetes and breast cancer within the United Arab Emirates (UAE), a subject of considerable global health concern. Given the increasing incidence of both diseases worldwide, this research investigates explicitly the potential influence of diabetes on the staging of breast cancer. The UAE, mirroring global trends, has experienced a surge in both conditions attributed to a blend of genetic, environmental, and lifestyle factors. The core objective of this investigation is to explore the link between diabetes and the stage at which breast cancer is diagnosed in UAE patients.

Material and method

To conduct this study, data were extracted from an extensive medical database consisting of anonymized records about breast cancer patients and their comorbid conditions. The research encompassed adult patients of all genders, all of whom had been definitively diagnosed with breast cancer. The data was analyzed using a suite of Python libraries, including Pandas, NumPy, SciPy, Scikit-learn, Matplotlib, and Seaborn. Descriptive and inferential statistical methods were employed, focusing on the Chi-Square test and logistic regression analysis to evaluate the relationship between diabetes and the stages of breast cancer, considering other comorbidities as well.

Results

The analysis included 131 breast cancer patients, predominantly female (98.47%), with an average age of 54.2 years. Among these patients, 22.14% were diabetic. The prevalence of other comorbidities, such as dyslipidemia, hypertension, and hypothyroidism, was also recorded. The Chi-Square test indicated no significant correlation between diabetes and the stages of breast cancer (χ² = 3.07, p = 0.381). Stage II was the most frequently diagnosed, irrespective of the presence or absence of diabetes.

Conclusion

In conclusion, this study finds no substantial link between diabetes and the stage of breast cancer diagnosis among patients in the UAE after adjusting for age and other comorbid conditions. These results underscore the need for early breast cancer detection approaches that are not exclusively dependent on the diabetic status of the patients. However, limitations such as the retrospective cohort design and the relatively small sample size highlight the necessity for further comprehensive studies. Such research would deepen the understanding of the relationship between diabetes and breast cancer and contribute to the advancement of breast cancer healthcare in the UAE.

## Introduction

Diabetes and breast cancer are two significant health challenges that affect millions of people worldwide. Both diseases are influenced by genetic, environmental, and lifestyle factors and have significant impacts on morbidity and mortality. The intricate relationship between diabetes and breast cancer has emerged as a critical area of study in medical research.

Diabetes, notably type 2, is a widespread chronic condition that has been linked to an increased risk of several cancers, including breast cancer [1,2]. It is a chronic metabolic disorder that results from insufficient insulin production or impaired insulin action, leading to high blood glucose levels. Uncontrolled diabetes can cause various complications, such as cardiovascular disease, nephropathy, neuropathy, and retinopathy. According to the International Diabetes Federation, the United Arab Emirates (UAE) had approximately one million people with diabetes in 2021, accounting for 16.4% of the adult population, and this number is projected to rise to 1.3 million by 2045 [3].

Breast cancer is the most common cancer among women worldwide and the leading cause of cancer-related death among women in the UAE [4]. Breast cancer is influenced by both modifiable and non-modifiable risk factors, such as age, family history, reproductive history, hormone therapy, obesity, physical activity, diet, alcohol consumption, and exposure to environmental carcinogens [5]. In the UAE, breast cancer has a unique epidemiological profile, as it tends to affect younger women, present at a later stage, and have a poorer prognosis than in others [6]. Moreover, the UAE faces several challenges in the prevention, diagnosis, and treatment of breast cancer, such as low awareness, cultural barriers, limited resources, and inadequate healthcare systems [7].

In the UAE, diabetes, and breast cancer have been increasing in prevalence and incidence over the past three decades due to rapid economic and social changes that have led to a lifestyle characterized by a high-calorie diet, obesity, and physical inactivity [8]. The causal link between diabetes and cancer is still under investigation, but recent studies have highlighted some of the possible pathways and consequences. Diabetes increases the risk of various cancers, such as breast cancer, by altering the hormonal, growth factor, and inflammatory milieu that modulates tumor initiation and growth [9-11]. Studies have shown that women with diabetes, particularly type 2, may have an elevated risk of developing breast cancer compared to those without diabetes [12]. This relationship is further complicated by factors such as hyperinsulinemia, obesity, and the metabolic alterations associated with diabetes [13,14]. These factors are thought to contribute to a pro-carcinogenic environment, potentially accelerating the initiation and progression of breast cancer. Moreover, the impact of diabetes on breast cancer extends beyond the risk of occurrence; it also encompasses aspects like prognosis, mortality, and the effectiveness of cancer treatments [15,16].

Therefore, diabetes and breast cancer are significant public health issues that require urgent attention and action in the UAE. There is a need for more research and data on the epidemiology, risk factors, and outcomes of these diseases, as well as the development and implementation of effective and culturally appropriate strategies to prevent, detect, and treat them. Furthermore, there is a need for more collaboration and coordination among the stakeholders, such as governments, health care providers, researchers, civil society, and patients, to address the burden of diabetes and breast cancer in the UAE. Given the global prevalence of both conditions, understanding the dynamics of this relationship is crucial for developing effective prevention and management strategies for breast cancer in diabetic patients [17].

The objective of the study was to investigate the association between diabetes and the stage of breast cancer at diagnosis within a diverse cohort of patients in the UAE. This research aimed to explore how diabetes, along with other demographic and clinical characteristics such as age, gender, nationality, and family history of cancer, may influence the progression and staging of breast cancer. Additionally, the study sought to contribute to understanding breast cancer dynamics in a multicultural setting, reflecting the unique demographic composition of the UAE.

## Materials and methods

Study design

This research is a retrospective cohort study conducted to investigate the association between diabetes mellitus (DM) and the stage of breast cancer at diagnosis, among other factors. We retrospectively analyzed medical records of patients diagnosed with breast cancer from 2007 to 2019. This time frame was chosen to encompass advancements in breast cancer diagnosis and treatment and changes in DM management, providing a comprehensive overview of their potential interaction over time.

Population and sample size

The study population consisted of patients diagnosed with breast cancer, identified from a tertiary care hospital's electronic health records (Tawam Hospital, UAE). Considering the exploratory nature of our study and the inherent limitations of retrospective data, we aimed to include as many eligible records as possible within the specified period.

Sample size calculation

Our initial calculation aimed to determine the sample size needed to estimate the proportion of breast cancer patients with DM with a 95% confidence level and a 4% margin of error, assuming the worst-case scenario for variance with a proportion (p) of 0.5. The formula used was:

n=(Z^2^×p×(1−p)/MOE^2^)

Z^2^ is the Z-score corresponding to a 95% confidence level (1.96), pp is the proportion (0.5), and MOE is the margin of error (0.04). Based on these parameters, the calculated sample size was approximately 375 patients. However, due to constraints related to the availability and completeness of medical records, the study ultimately included 131 patients. We acknowledge this limitation and discuss its implications in the analysis and interpretation of our findings.

Data collection

Data were collected from the hospital's electronic health records based on predefined inclusion criteria. All patients were diagnosed with breast cancer within the study period, with no restrictions on age or stage at diagnosis. The collected data encompassed: Demographic Information: Age at diagnosis, gender, and nationality; Clinical Data: Presence of DM, dyslipidemia, hypertension, hypothyroidism, congestive heart failure (CHF), chronic kidney disease (CKD), rheumatoid arthritis (RA); Breast Cancer Details: Type of breast cancer, stage at diagnosis (utilizing the TNM classification), year of diagnosis, and family history of breast cancer. Only complete records were included, and patients with missing data were omitted from the study.

Data analysis

The statistical analysis plan for this study was designed to methodically explore the relationship between DM and breast cancer stage at diagnosis, alongside other relevant demographic and clinical variables. The analysis was conducted in several stages to ensure a comprehensive examination of the data:

Descriptive statistics

Initially, we performed descriptive statistics to summarize the characteristics of the study cohort, stratified by the presence or absence of DM. This included calculating means and standard deviations for continuous variables (e.g., age) and frequencies and percentages for categorical variables (e.g., nationality, type of breast cancer, stage of breast cancer). This step provided a foundational understanding of key variables' distribution and central tendencies within our sample, facilitating a comparison between the two subgroups (DM vs. no DM) and the total cohort.

Univariate analysis

Following the descriptive analysis, univariate analyses were conducted to assess the association between each study variable (including DM) and the outcome variable (breast cancer stage). The choice of statistical tests depended on the nature of the variables being analyzed: For dichotomous variables (e.g., presence of hypertension, family history of breast cancer), Chi-square tests or Fisher's exact tests were used, depending on the expected frequencies in the cross-tabulations. This step aimed to identify variables that showed a significant association with breast cancer stage, which could warrant further investigation in the multivariate analysis.

Multivariate logistic regression model

The final stage of our analysis involved constructing a multivariate logistic regression model to explore the independent effect of DM on the stage of breast cancer at diagnosis, controlling for other significant variables identified in the univariate analysis. Variables entered into the model included:

Demographic Factors

Age, as a continuous variables, and gender, if applicable.

Clinical Variables

The presence of DM, dyslipidemia, hypertension, hypothyroidism, CHF, CKD, RA, and any other variables showed a significant association with breast cancer stage in the univariate analysis.

The selection of variables for the multivariate model was based on their statistical significance in the univariate analysis, their clinical relevance to breast cancer stage or diabetes, and considerations of multicollinearity. This approach ensured that the model included factors most likely to influence the outcome, providing a nuanced understanding of the relationship between DM and breast cancer stage and adjusted for potential confounders.

We adopted a strategic approach to ensure a robust and meaningful analysis for the logistic regression addressing the Nationality variable. Recognizing the diverse representation of nationalities within our study population and the need for statistical validity, we categorized nationalities based on the frequency of occurrence within our dataset. Specifically, nationalities represented by five or more participants were individually categorized and analyzed. This criterion ensured that each categorized nationality had a sufficient sample size to contribute to meaningful statistical analysis and interpretation. To accommodate our cohort's wide variety of nationalities, yet not individually meeting the threshold of five participants, we introduced a consolidated category termed "Others." This category encompasses all nationalities with fewer than five participants, thereby allowing for their inclusion in the analysis without compromising the results' statistical robustness or interpretability. This approach adheres to best data management and statistical analysis practices and reflects the demographic diversity intrinsic to our study population. This methodological decision enables a focused analysis of the most frequently represented nationalities while maintaining inclusivity and respecting the demographic complexity of our cohort. It aligns with our study's objectives to provide statistically valid and demographically comprehensive insights, facilitating an understanding of any nationality-specific trends or associations within the context of our research objectives.

The logistic regression results were presented as odds ratios (ORs) with 95% confidence intervals (CIs), indicating the strength and direction of the associations between each independent variable and the outcome. A p-value of <0.05 was considered statistically significant.

Data analysis was conducted using RStudio, Tableau, Python, and its libraries. Heatmaps were generated to visually represent the distribution of breast cancer stages among patients with and without diabetes. These visualizations aided in identifying patterns and trends in the data.

Ethical considerations

The study complied with ethical standards approved by the Tawam Human Research Ethics Committee (THREC) number MF2024-661, with all patient data anonymized and confidentiality maintained.

## Results

**Figure 1:**
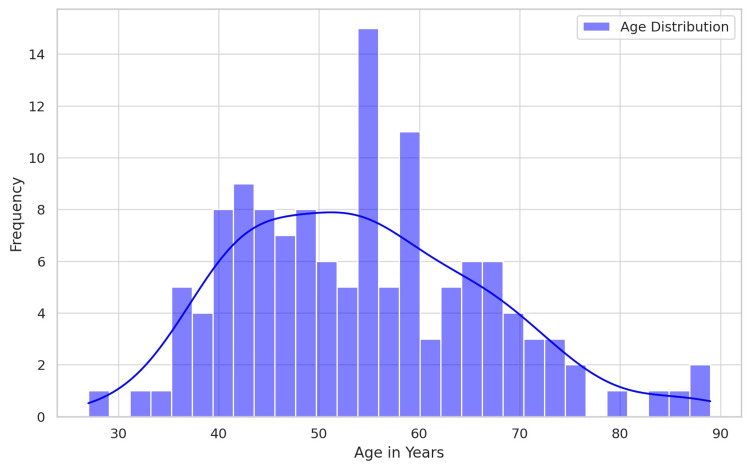
Age distribution N=131

The study examined a group of 131 individuals diagnosed with breast cancer, with their ages ranging from 27 to 89 years. The mean age was 54.2 years, with a standard deviation of 12.35 (Figure 1).

Most of these patients were female, comprising 98.47% of the group (129 females), and only 1.53% were male (two males). Regarding diabetes prevalence, 22.14% of the patients (29 individuals) had diabetes, while 77.86% (102 individuals) did not. Furthermore, a notable 35.88% of the patients (47 individuals) had a family history of cancer (Table 1).

**Table 1:** Descriptive statistics of breast cancer patients' demographic and clinical characteristics CHF: Congestive heart failure; CKD: Chronic kidney disease; RA: Rheumatoid arthritis; std: standard deviation; N=131

Age in Years	Mean	std	Min	Max	25th	50th	75th
	54.2	12.35	27	89	44.5	54	89
Gender	Female (%)	Male (%)					
	129 (98.47)	02 (1.53)					
Nationality	Emirati	Egyptian	Syrian	Filipino	Pakistani		
n (%)	38 (29.01)	15 (11.45)	14 (10.69)	10 (7.63)	9 (6.87)		
Comorbidities	No (%)	Yes (%)					
Diabetes	102 (77.86)	29 (22.14)					
Dyslipidemia	117 (89.31)	14 (10.69)					
Hypertension	91(69.47)	40 (30.53)					
Hypothyroid	121 (92.37)	10 (7.63)					
CHF	129 (98.47)	2 (1.53)					
CKD	130 (99.24)	1 (0.76)					
RA	130 (99.24)	1 (0.76)					
Family History of Cancer	84 (64.12)	47 (35.88)

**Figure 2:**
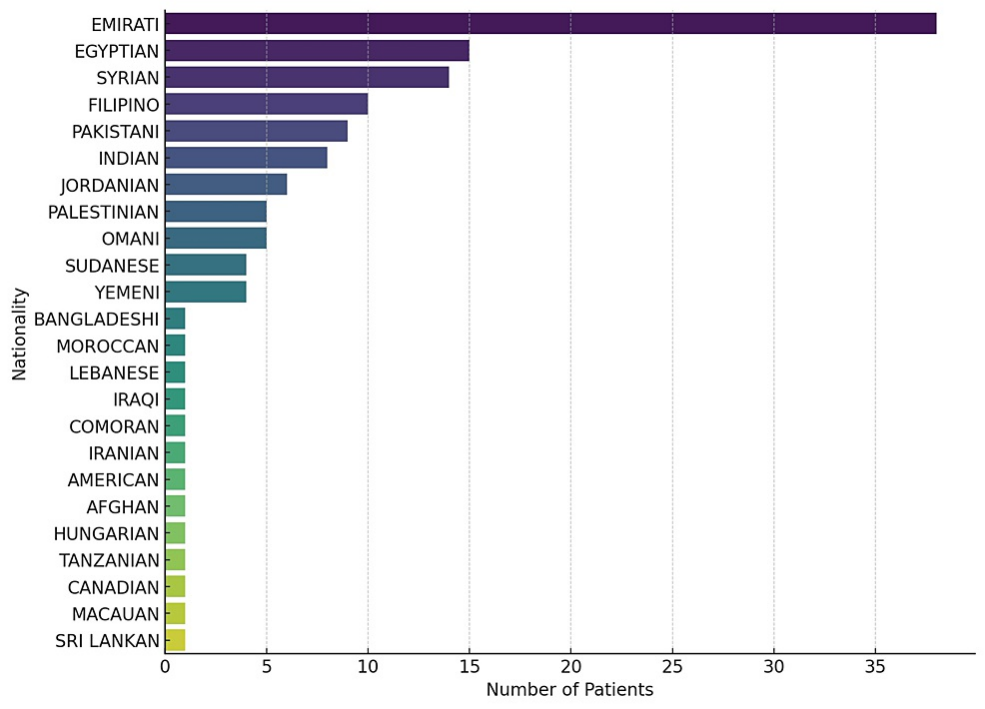
Various nationalities of the patients in this study N=131

Regarding nationality among the 131 patients in the study, the largest group was Emirati, representing 29.01% of the sample (38 Emirati patients). This was followed by Egyptian nationals, who accounted for 11.45% of the cohort (15 Egyptian patients), Syrian nationals at 10.69% (14 Syrian patients), Filipino nationals comprising 7.63% (10 Filipino patients), and Pakistani nationals making up 6.87% (nine Pakistani patients). The remaining 34.35% of the sample consisted of other nationalities, amounting to 45 patients from different nationalities (Figure 2).

The Chi-Square Test of Independence revealed no statistically significant association between diabetes and breast cancer stages (χ² = 3.07, df = 3, p = 0.381). This indicates that there was no statistically significant association between diabetes and the stages of breast cancer in our patient cohort, as the p-value did not meet the conventional threshold for statistical significance of p < 0.05 (Table 2).

**Table 2:** Chi-square tests for each categorical variable and outcome variable (stage of breast cancer) DF: degrees of freedom; CHF: congestive heart failure; CKD: chronic kidney disease; RA: rheumatoid arthritis;

Test	Chi-square	DF	P-Value	Decision
Diabetes	3.07	3	0.381	no association
Nationality	76.23	69	0.2573	no association
Dyslipidaemia	0.83	3	0.8425	no association
Hypertension	1.03	3	0.7942	no association
Hypothyroid	6.77	3	0.0796	no association
CHF	3.84	3	0.2796	no association
CKD	4.95	3	0.1757	no association
RA	3.36	3	0.3395	no association
Type of cancer	14.11	18	0.7217	no association
Family History of Cancer	2.56	3	0.4637	no association

**Figure 3:**
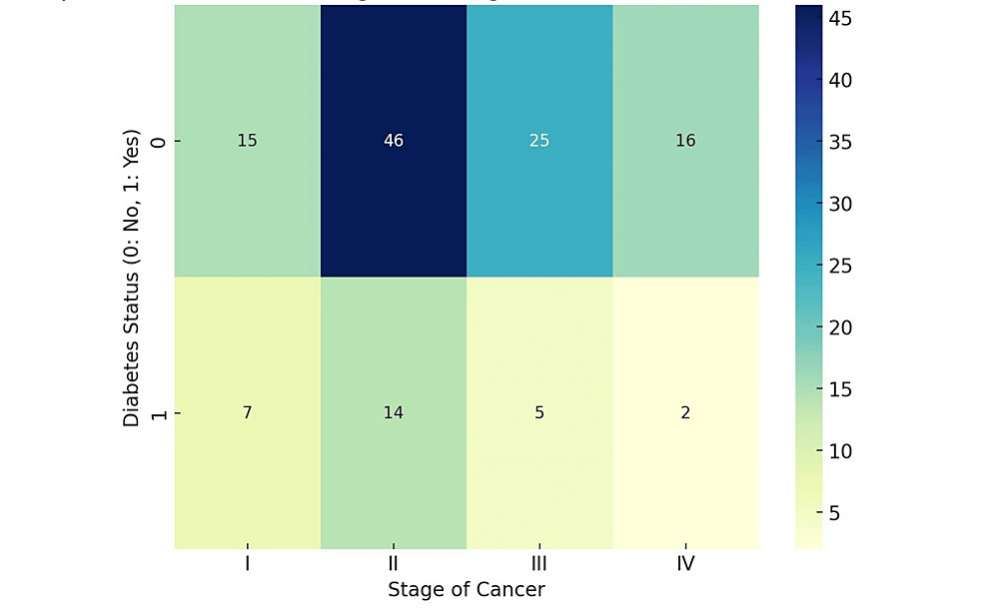
Diabetes and stage of breast cancer

**Figure 4:**
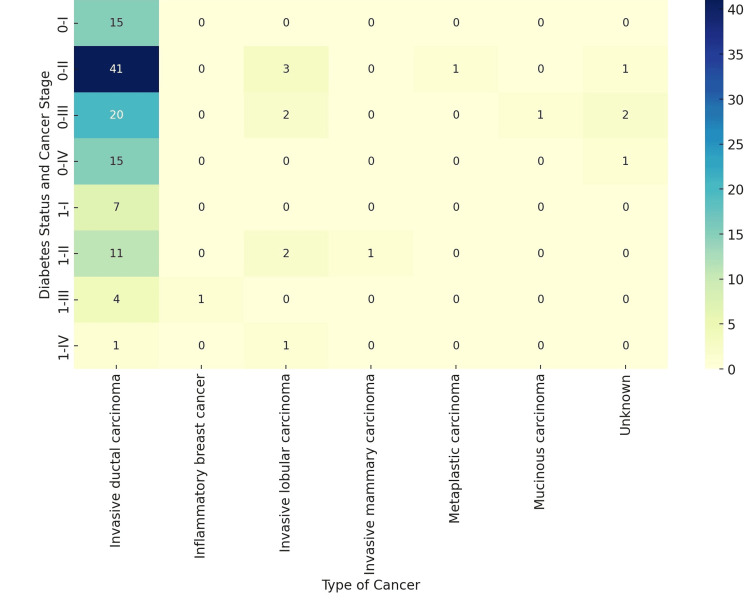
Diabetes, breast cancer stage and histopathology of the cancer 1: Diabetes present, 0: Diabetes absent; I, II, III, IV: breast cancer stages; so 0-I must be read as non-diabetic stage 1 breast cancer.

Heatmaps were used to visualize the distribution of breast cancer stages among patients with and without diabetes. These visualizations highlighted that most cases, irrespective of diabetes status, were classified as Stage II Invasive Ductal Carcinoma (Figures 3, 4).

The logistic regression analysis, aimed at evaluating the impact of diabetes on breast cancer stage while adjusting for age and comorbidities, indicated a notable association with an 82% reduction in breast cancer odds for diabetic individuals (OR=0.18, 95% CI=0.03-0.95, p=0.043). Despite an overall model accuracy of 46%, the analysis showed variability in predictive effectiveness across stages, particularly exhibiting low precision and recall for stages I and IV. Notably, stage II breast cancer demonstrated the highest recall rate of 0.89, highlighting enhanced model performance for this stage (Table 3).

**Table 3:** Associations between demographic, clinical characteristics, and the likelihood of breast cancer stage: logistic regression analysis Intercept: The model's constant term, representing the log odds of the outcome when all predictors are held at zero; Age in Years: The continuous variable representing the age of the participants; Emirati: Refers to participants of Emirati nationality; Filipino: Refers to participants of Filipino nationality; Indian: Refers to participants of Indian nationality; Jordanian: Refers to participants of Jordanian nationality; Omani: Refers to participants of Omani nationality; Others: A consolidated category for nationalities represented by fewer than five participants in the study; Pakistani: Refers to participants of Pakistani nationality; Palestinian: Refers to participants of Palestinian nationality; Syrian: Refers to participants of Syrian nationality; Diabetes: Indicates the presence of diabetes mellitus; Dyslipidemia: Indicates the presence of dyslipidemia; Hypertension: Indicates the presence of hypertension; Hypothyroid: Indicates the presence of hypothyroidism; Family History: Indicates a family history of breast cancer; Odds Ratio (OR): This represents the odds of the outcome occurring (e.g., stage of breast cancer) for participants with the variable, compared to the odds for those without the variable; Lower CI (Confidence Interval) and Upper CI: The 95% confidence interval for the odds ratio, indicating the range within which the true odds ratio is likely to fall; p-values: The probability of observing the data, or something more extreme, if the null hypothesis of no association is true. A p-value less than 0.05 is typically considered statistically significant.

	Odds Ratio	lower ci	upper ci	p-values
(Intercept)	209.68	6.27	7016.60	0.002840
Age in years	0.95	0.91	1.00	0.055059
Emirati	0.31	0.04	2.17	0.238890
Filipino	0.52	0.03	8.69	0.645980
Indian	0.23	0.02	2.90	0.257972
Jordanian	71686858.32	0.00	Inf	0.996282
Omani	0.52	0.03	10.05	0.662823
Others	0.39	0.05	3.15	0.377331
Pakistani	0.54	0.05	6.24	0.618851
Palestinian	39369644.62	0.00	Inf	0.996767
Syrian	0.41	0.05	3.49	0.416338
Diabetes	0.18	0.03	0.95	0.043236
Dyslipidemia	2.37	0.21	26.39	0.481384
Hypertension	2.35	0.47	11.74	0.297456
Hypothyroid	87785192.26	0.00	Inf	0.995251
Family History of Cancer	0.30	0.09	1.00	0.050513

## Discussion

Several mechanisms have been proposed to explain how diabetes may influence breast cancer development and progression, such as hyperinsulinemia, hyperglycemia, chronic inflammation, oxidative stress, and altered levels of sex hormones and adipokines [18]. These factors may stimulate the growth and survival of breast cancer cells and enhance their invasiveness and metastatic potential. Moreover, diabetes may interfere with the efficacy and toxicity of breast cancer therapies, such as chemotherapy, hormonal therapy, and targeted therapy. Therefore, diabetes may have a complex and multifaceted impact on breast cancer outcomes, depending on the type, duration, and severity of diabetes, as well as the stage, subtype, and treatment of breast cancer.

The present study also revealed that the most common stage of breast cancer among patients in the UAE was stage II, followed by stage III, irrespective of diabetes status. This finding indicates that breast cancer is diagnosed at relatively late stages in the region, which may be attributed to several factors, such as low awareness, lack of screening programs, cultural barriers, and limited access to health care services. Early detection of breast cancer is crucial for improving the survival and quality of life of patients, as well as reducing the cost and burden of treatment [19].

This study explored the relationship between diabetes and breast cancer stages, revealing no statistically significant association (χ² = 3.07, df = 3, p = 0.381). This finding aligns with several studies indicating a complex interplay between diabetes and breast cancer. Lipscombe et al. reported a significant association between diabetes and advanced-stage breast cancer, suggesting that diabetes may predispose to more aggressive forms of the disease [20]. Alenzi et al. also observed a correlation between the severity of diabetes complications and advanced stages of breast cancer, emphasizing the potential impact of diabetes management on cancer outcomes [21]. However, contrasting findings are noted in the literature. Jousheghany et al. found no significant association between HbA1C levels and tumor stage among breast cancer patients, highlighting the nuanced nature of this relationship [22].

Moreover, the chi-square test analysis in this study showed limited effectiveness of diabetes in predicting certain stages of breast cancer. This diverges from the findings of Chen et al., who noted a reduced risk of adverse breast cancer outcomes with metformin use, indicating the potential benefits of specific diabetic treatments [23]. These mixed results in the literature underscore the complexity of the interaction between diabetes and breast cancer and the need for further research in this area.

The UAE presents a unique context for examining breast cancer, given its diverse population composition and the prevalence of the disease. A significant expatriate presence alongside the local Emirati population characterizes the UAE's population. Jamal highlights that the local population constitutes less than 11.5% of the total population, with a substantial number of expatriates from various regions​​ [24]. This study's patient cohort then adequately represents the population in line with the studies in the literature. Understanding the unique demographic composition and health behaviors in the UAE is essential for tailoring public health initiatives and medical interventions to address breast cancer in this region effectively. This approach should encompass cultural sensitivities, varied levels of awareness, and access to healthcare across different population groups.

The study has several strengths, such as being the first to investigate the association between diabetes and the stage of breast cancer in the UAE, using a standardized and validated tool (American Joint Commission Staging Manual) to assess the stage of breast cancer, and adjusting for potential confounders in the analysis [25].

Limitations

However, the study also has some limitations that should be acknowledged. First, the study was based on a retrospective design, which precludes the establishment of causal inference between diabetes and breast cancer stage. Second, the study did not include some relevant variables, such as the duration and treatment of diabetes, lifestyle, and environmental factors that may affect both diseases and could be included in subsequent studies. Finally, the study was conducted in a single center, which may not represent the diversity and heterogeneity of the region.

## Conclusions

This study meticulously explored the demographic and clinical characteristics of 131 individuals diagnosed with breast cancer, revealing a diverse cohort with a mean age of 54.2 years, predominantly female. The investigation into the prevalence of diabetes among these patients showed that 22.14% had diabetes, highlighting the significance of understanding the interplay between chronic conditions and cancer outcomes. Furthermore, the distribution of nationalities within the study population, with Emirati nationals forming the largest group, reflects the cultural and demographic diversity of the UAE, providing a unique context for examining breast cancer characteristics. Despite the exploration of various factors, including a family history of cancer and diabetes status, our findings, through the application of the Chi-Square Test of Independence, did not demonstrate a statistically significant association between diabetes and breast cancer stages, suggesting that within this cohort, diabetes does not influence the stage at which breast cancer is diagnosed.

Utilizing heatmaps to visualize the distribution of breast cancer stages among patients, particularly noting a predominance of Stage II Invasive Ductal Carcinoma, highlights the potential of advanced data visualization techniques in enhancing our understanding of cancer epidemiology. While the absence of a statistically significant association between diabetes and breast cancer stage in our study suggests that diabetes may not be a determining factor in the progression of breast cancer within this population, it opens avenues for future research. Investigations focusing on the impact of residency duration, lifestyle, and environmental factors prevalent before migration to the UAE and exploring other comorbidities and genetic predispositions could offer deeper insights. Our findings contribute to the growing body of literature on breast cancer, emphasizing the need for continued research in diverse populations to tailor prevention and treatment strategies effectively.

## References

[1] Larsson SC, Mantzoros CS, Wolk A (2007). Diabetes mellitus and risk of breast cancer: a meta-analysis. Int J Cancer.

[2] Eketunde AO (2020). Diabetes as a Risk Factor for Breast Cancer. Cureus.

[3] Saeedi P, Petersohn I, Salpea P (2019). Global and regional diabetes prevalence estimates for 2019 and projections for 2030 and 2045: Results from the International Diabetes Federation Diabetes Atlas, 9(th) edition. Diabetes Res Clin Pract.

[4] Safiri S, Noori M, Nejadghaderi SA (2022). Burden of female breast cancer in the Middle East and North Africa region, 1990-2019. Arch Public Health.

[5] Zhao P, Xia N, Zhang H, Deng T (2020). The Metabolic Syndrome Is a Risk Factor for Breast Cancer: A Systematic Review and Meta-Analysis. Obes Facts.

[6] (2024). UAE National Cancer Registry. Cancer Incidence in the United Arab Emirates: Annual Report of the UAE [Online]. https://mohap.gov.ae/assets/download/bc820447/CANCER%20INCIDENCE%20IN%20UNITED%20ARAB%20EMIRATES%20ANNUAL%20REPORT%20OF%20THE%20UAE%20-%202019.pdf.aspx..

[7] Fayed R, Hamza D, Abdallah H, Kelany M, Tahseen A, Aref AT (2017). Do we need regional guidelines for breast cancer management in the MENA region? MENA Breast Cancer Guidelines project. Ecancermedicalscience.

[8] Mansour R, Al-Ani A, Al-Hussaini M, Abdel-Razeq H, Al-Ibraheem A, Mansour AH (2024). Modifiable risk factors for cancer in the middle East and North Africa: a scoping review. BMC Public Health.

[9] Wolf I, Sadetzki S, Catane R, Karasik A, Kaufman B (2005). Diabetes mellitus and breast cancer. Lancet Oncol.

[10] Tobe A, Horimoto Y, Kobayashi K, Kamisada N, Hirano M (2022). Impact of Diabetes on Patient Outcomes in Breast Cancer Patients. Breast Care (Basel).

[11] Giovannucci E, Harlan DM, Archer MC (2010). Diabetes and cancer: a consensus report. Diabetes Care.

[12] Ferroni P, Riondino S, Buonomo O, Palmirotta R, Guadagni F, Roselli M (2015). Type 2 Diabetes and Breast Cancer: The Interplay between Impaired Glucose Metabolism and Oxidant Stress. Oxid Med Cell Longev.

[13] Martin SD, McGee SL (2018). Metabolic reprogramming in type 2 diabetes and the development of breast cancer. J Endocrinol.

[14] Li CI, Daling JR, Tang MT, Malone KE (2011). Relationship between diabetes and risk of second primary contralateral breast cancer. Breast Cancer Res Treat.

[15] Jiralerspong S, Kim ES, Dong W, Feng L, Hortobagyi GN, Giordano SH (2013). Obesity, diabetes, and survival outcomes in a large cohort of early-stage breast cancer patients. Ann Oncol.

[16] Lega IC, Lipscombe LL (2020). Review: Diabetes, Obesity, and Cancer-Pathophysiology and Clinical Implications. Endocr Rev.

[17] Resta F, Triggiani V, Sabbà C (2004). The impact of body mass index and type 2 diabetes on breast cancer: current therapeutic measures of prevention. Curr Drug Targets Immune Endocr Metabol Disord.

[18] Barone BB, Yeh HC, Snyder CF (2008). Long-term all-cause mortality in cancer patients with preexisting diabetes mellitus: a systematic review and meta-analysis. JAMA.

[19] El Saghir NS, Seoud M, Khalil MK, Charafeddine M, Salem ZK, Geara FB, Shamseddine AI (2006). Effects of young age at presentation on survival in breast cancer. BMC Cancer.

[20] Lipscombe LL, Fischer HD, Austin PC (2015). The association between diabetes and breast cancer stage at diagnosis: a population-based study. Breast Cancer Res Treat.

[21] Alenzi EO, Madhavan SS, Tan X (2018). Association of the severity of diabetes-related complications with stage of breast cancer at diagnosis among elderly women with pre-existing diabetes. Breast Cancer Res Treat.

[22] Jousheghany F, Phelps J, Crook T, Hakkak R (2015). Relationship Between Level of HbA1C and Breast Cancer Outcomes.. The FASEB Journal.

[23] Lega IC, Fung K, Austin PC, Lipscombe LL (2017). Metformin and breast cancer stage at diagnosis: a population-based study. Curr Oncol.

[24] Jamal Jamal, Manal A (2015). “The ‘Tiering’ of Citizenship and Residency and the ‘Hierarchization’ of Migrant Communities: The United Arab Emirates in Historical Context.” The International Migration Review. JSTOR.

[25] Byrd DR, Brookland RK, Washington MK (2017 Jan). AJCC cancer staging manual. https://link.springer.com/book/9783319406176?utm_medium=email&utm_source=transaction.

